# Hydrogen-rich water reduces inflammatory responses and prevents apoptosis of peripheral blood cells in healthy adults: a randomized, double-blind, controlled trial

**DOI:** 10.1038/s41598-020-68930-2

**Published:** 2020-07-22

**Authors:** Minju Sim, Chong-Su Kim, Woo-Jeong Shon, Young-Kwan Lee, Eun Young Choi, Dong-Mi Shin

**Affiliations:** 1 Department of Food and Nutrition, Seoul National University, Seoul, 08826 Republic of Korea; 2 Department of Biomedical Sciences, Seoul National University College of Medicine, Seoul, 03080 Republic of Korea; 3 Research Institute of Human Ecology, Seoul National University, Seoul, 08826 Republic of Korea

**Keywords:** Cell biology, Molecular biology

## Abstract

The evidence for the beneficial effects of drinking hydrogen-water (HW) is rare. We aimed to investigate the effects of HW consumption on oxidative stress and immune functions in healthy adults using systemic approaches of biochemical, cellular, and molecular nutrition. In a randomized, double-blind, placebo-controlled study, healthy adults (20–59 y) consumed either 1.5 L/d of HW (*n* = 20) or plain water (PW, *n* = 18) for 4 weeks. The changes from baseline to the 4th week in serum biological antioxidant potential (BAP), derivatives of reactive oxygen, and 8-Oxo-2′-deoxyguanosine did not differ between groups; however, in those aged ≥ 30 y, BAP increased greater in the HW group than the PW group. Apoptosis of peripheral blood mononuclear cells (PBMCs) was significantly less in the HW group. Flow cytometry analysis of CD4^+^, CD8^+^, CD20^+^, CD14^+^ and CD11b^+^ cells showed that the frequency of CD14^+^ cells decreased in the HW group. RNA-sequencing analysis of PBMCs demonstrated that the transcriptomes of the HW group were clearly distinguished from those of the PW group. Most notably, transcriptional networks of inflammatory responses and NF-κB signaling were significantly down-regulated in the HW group. These finding suggest HW increases antioxidant capacity thereby reducing inflammatory responses in healthy adults.

## Introduction

Oxidative stress indicates a state where excessive reactive oxygen species (ROS) overwhelm the biological antioxidant capacity, leading to disruption of ROS homeostasis and cellular damage^1^. It is important for cells to maintain moderate levels of ROS to perform normal physiological functions^2^. Excessive level of ROS are responsible for oxidative damage of DNA and lipids, which may lead to cellular death^3^. Also, oxidative stress may provoke inflammatory responses^3,4^ that can further enhance oxidative stress. As a result, oxidative stress can act to precipitate chronic inflammation, with pathological conditions triggering various disorders including cardiovascular diseases, metabolic syndrome, neurodegenerative disorders, and cancer^5–8^.

There is no doubt that oxidative stress plays a central role in the pathogenesis of various chronic diseases. As a result, it has been of increasing interest to assess adjuvant effects of antioxidant agents in food on prevention and alleviation of these diseases. Recently, the US Food and Drug Administration acknowledged hydrogen (H_2_) gas as food additives when used in drinking water or beverages and declared them to be generally recognized as safe. H_2_ can be a novel antioxidant because of its ability to selectively scavenge strong oxidants such as hydroxyl radical^9^. In models of ischemia/reperfusion injury, H_2_ prevented tissue damage and reduced infarct size^10–12^. In rat models of neurodegenerative disorders, including Parkinson’s and Alzheimer’s diseases, administration of H_2_ improved the memory function of rats and retarded the progression of disease^13,14^. Some clinical trials have also determined the effect of H_2_ on the several diseases including metabolic syndrome, rheumatoid arthritis, chronic hepatitis B and Parkinson’s disease^15–18^.

Despite the increasing evidence attesting to the beneficial effects of H_2_, to our knowledge, few studies have been conducted in a healthy population. Furthermore, the systemic effect of H_2_ administration has not been elucidated because most of the preceding studies have only focused on measuring limited markers. Here, we aimed to investigate the effects of H_2_-rich water (HW) consumption in healthy adults through the extensive analyses of antioxidant capacity, peripheral blood mononuclear cell (PBMC) subsets and their transcriptome profile and to compare the effects of HW consumption with those of plain water (PW) consumption.

## Results

### Participants and baseline characteristics

**Figure 1:**
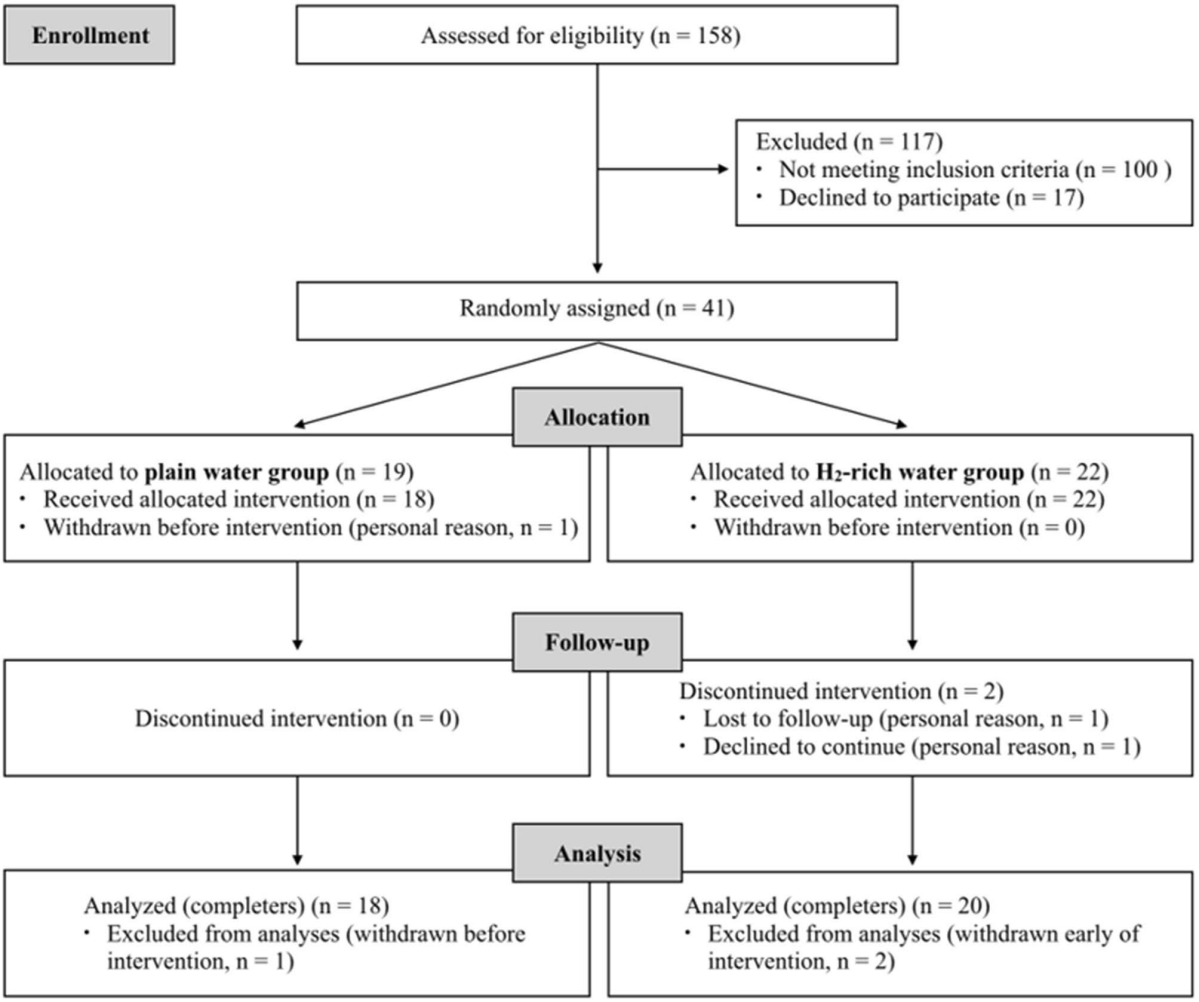
Flow diagram of the participants throughout the study.

The flow diagram of the participants throughout the study is presented in Fig. 1. A total of 158 participants were assessed for eligibility according to the inclusion and exclusion criteria. 41 participants were found to be eligible and were included in the study. They were randomly assigned to either the PW group (*n* = 19) or the HW group (*n* = 22). Out of 3 participants who withdrew from the study, 1 participant in PW group dropped out before starting the intervention, and 2 participants in HW group dropped out on the 4th day and the 10th day. As a result, a total of 38 participants successfully completed the 4-week intervention and were included in the final analysis (*n* = 18 in PW group; *n* = 20 in HW group) (Fig. 1).

As shown in Table 1, there were no statistical differences in age, height, weight, BMI and daily plain water intake at baseline between the PW and HW groups (all *P* > 0.05).

**Table 1:** General characteristics of participants at baseline.

Characteristics	PW group	HW group	*P*
Subjects (n)	18	20	–
Sex (M/F, n)	9/9	10/10	–
Age (y)	32.9 ± 10.9	29.6 ± 8.1	0.393
Height (cm)	167.6 ± 7.4	169.0 ± 9.1	0.602
Weight (kg)	66.5 ± 12.6	68.8 ± 15.7	0.493
BMI (kg/m^2^)	23.1 ± 2.7	23.8 ± 3.9	0.530
Daily plain water intake (L/d)	1.2 ± 0.5	1.2 ± 0.3	0.393

All values are means ± SDs. There were no statistical differences between the plain water (PW) group and the H_2_-rich water (HW) group on the basis of an unpaired *t* test or a Mann–Whitney *U* test. Information on daily plain water intake was obtained from self-reported questionnaires at baseline.

### Antioxidant capacity and oxidative damages

**Figure 2:**
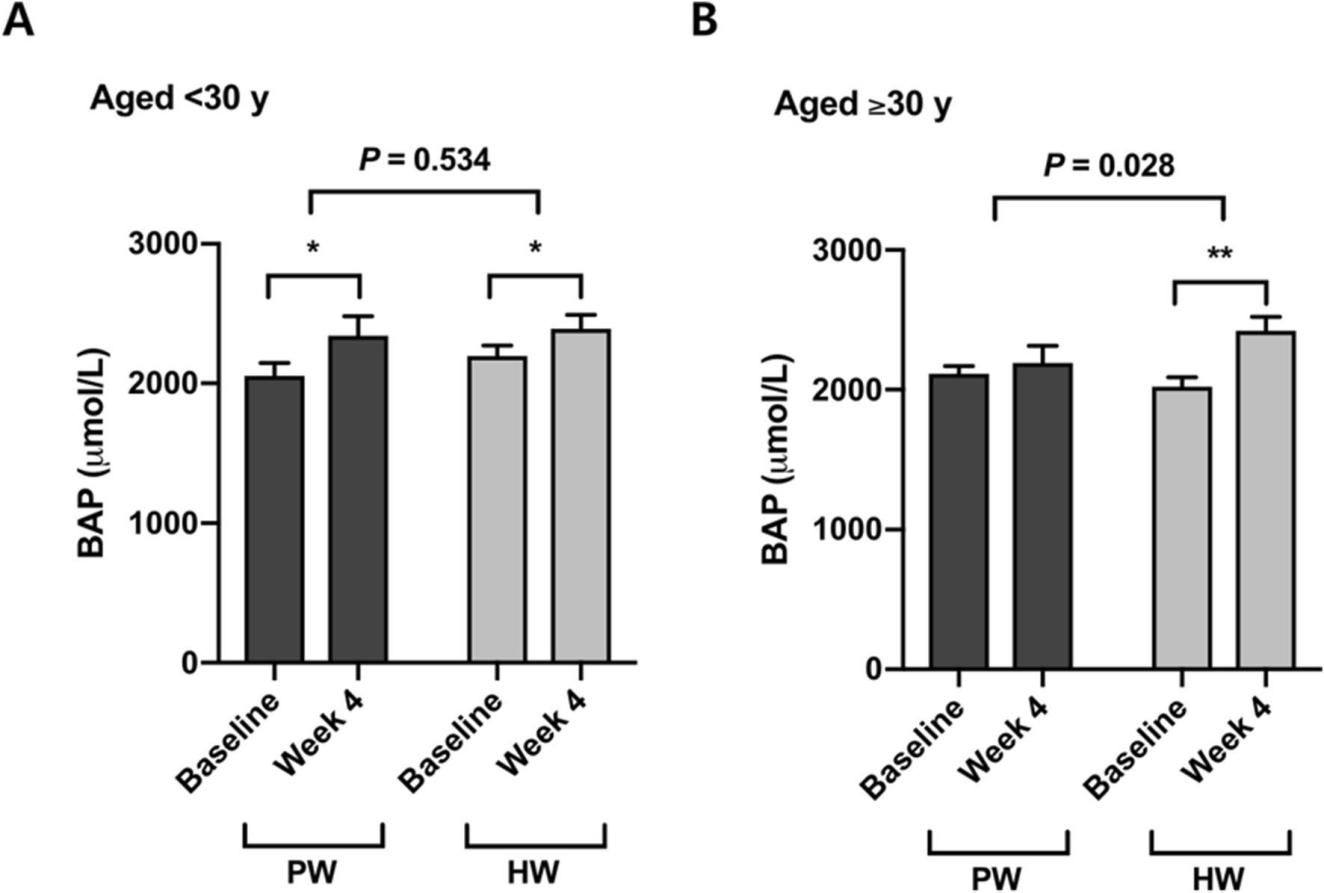
Changes from baseline in serum BAP by age (< 30 y and ≥ 30 y). Data are presented as means ± SEMs. Significant differences between baseline and week 4 within each group were determined with the use of a paired *t* test. *P* values were obtained with the use of simple main effects analysis and *P* < 0.05 was considered statistically significant. (**A**) Within the participants aged < 30 y, there was no significant difference between PW group (*n* = 10) and HW group (*n* = 10) for the change in BAP (*P* = 0.534). (**B**) HW group aged ≥ 30 y (*n* = 10) showed a greater increase in BAP compared with PW group aged ≥ 30 y (*n* = 8) (*P* = 0.028). PW, plain water; HW, H_2_-rich water; BAP, biological antioxidant potential.

Four-week consumption of both plain water and hydrogen-rich water increased serum biological antioxidant potential (BAP) (Δ = 194.4 ± 315.4 μmol/L, *P* < 0.05 in PW; Δ = 297.8 ± 274.2 μmol/L, *P* < 0.001 in HW) (Table 2). Although there was no significant difference in the between-group comparison of PW versus HW in the total population (*P* = 0.267) (Table 2), participants who were over 30 yrs old showed a significant increase in BAP by drinking hydrogen-rich water but not plain water, and the difference in the changes was significant (*P* = 0.028) (Fig. 2). On the contrary, no significant effect of hydrogen rich water on BAP was found in the younger group (< 30 y) (*P* = 0.534) (Fig. 2).

**Table 2:** Antioxidant capacity and oxidative damage markers.

Measure	PW group (*n* = 17–18)			HW group (*n* = 16–20)			PW versus HW
	Baseline	Week 4	∆	Baseline	Week 4	∆	*P*
BAP (µmol/L)	2,080.6 ± 236.6	2,275.0 ± 394.5	194.4 ± 315.4*	2,109.5 ± 234.5	2,407.3 ± 303.6	297.8 ± 274.2***	0.267
d-ROMs (CARR.U)	354.9 ± 70.7	349.0 ± 56.1	– 5.9 ± 28.9	391.5 ± 98.8	384.3 ± 96.4	– 7.3 ± 38.9	0.700
8-OHdG (ng/mL)	1.99 ± 1.27	1.04 ± 0.59	– 0.94 ± 1.44*	2.05 ± 0.95	0.73 ± 0.60	– 1.32 ± 1.05***	0.144

All values are means ± SDs. There were no significant differences between PW and HW groups for all measures at baseline on the basis of an unpaired *t* test or a Mann–Whitney *U* test. ∆ indicates the change from baseline to week 4. Significant differences between baseline and week 4 within each group were determined with the use of a paired *t* test (*P < 0.05; **P < 0.01; ***P < 0.001). *P* values were obtained with the use of a general linear model adjusting for the value at baseline as a covariate. PW, plain water; HW, H_2_-rich water; BAP, biological antioxidant potential; d-ROMs, derivatives of reactive oxygen metabolites; 8-OHdG, 8-Oxo-2-deoxyguanosine.

Oxidative stress in serum assessed by the level of derivatives of reactive oxygen (d-ROMs) was not affected by the 4-week intervention (all *P* > 0.05) (Table 2). The levels of 8-Oxo-2′-deoxyguanosine (8-OHdG), a marker for DNA damage, significantly decreased in both groups (Δ = − 0.94 ± 1.44 ng/mL, *P* < 0.05 in PW; Δ = − 1.32 ± 1.05 ng/mL, *P* < 0.001 in HW), but with no statistical difference between the PW and HW groups (all *P* > 0.05) (Table 2).

### Apoptosis of PBMCs and blood immune cell population profiles

**Figure 3:**
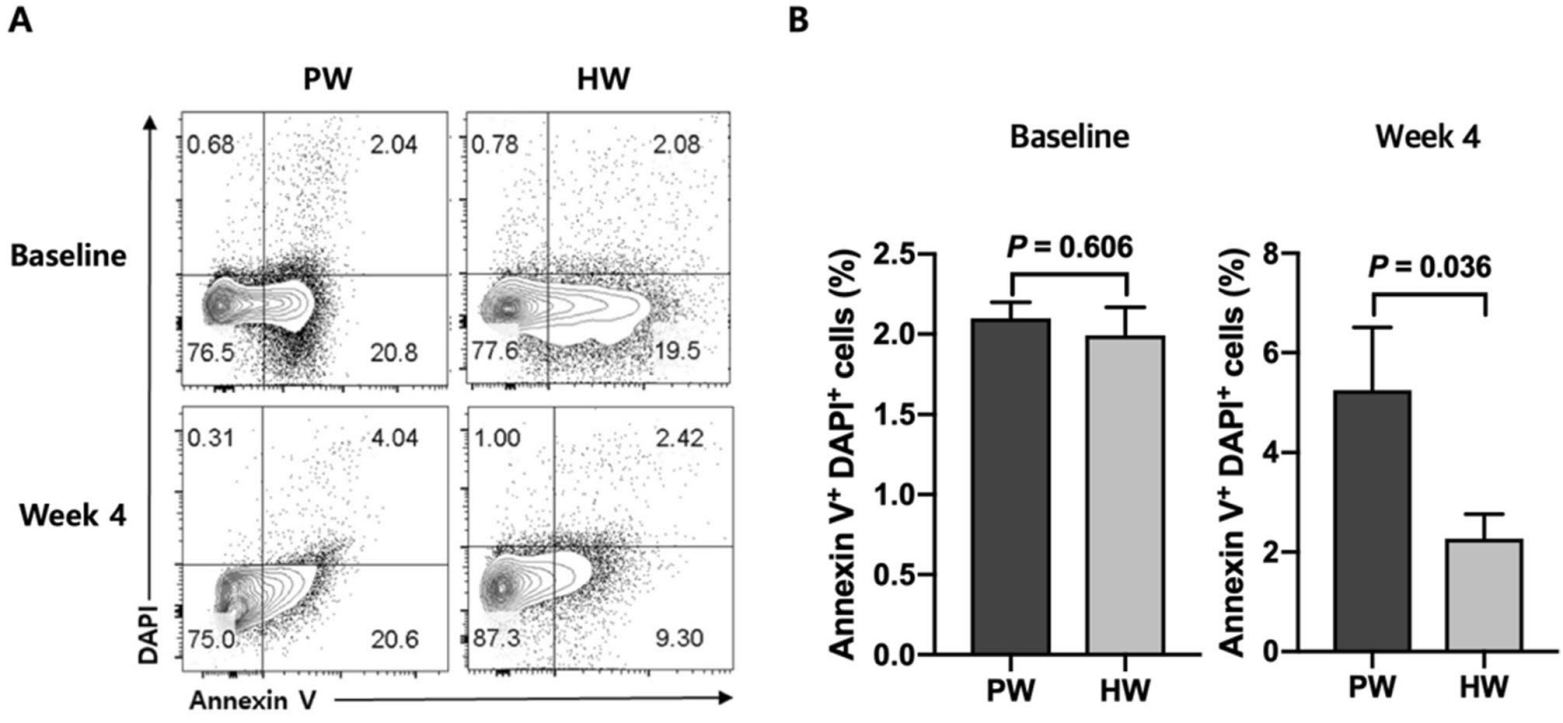
Representative flow cytometric data (**A**) and frequencies of apoptotic cells (Annexin V^+^ DAPI^+^) at baseline and week 4 (**B**). Data are presented as means ± SEMs. Significant differences between PW group (*n* = 14) and HW group (*n* = 15) at baseline were determined with the use of an unpaired *t* test or a Mann–Whitney *U* test, and those at week 4 were determined with a general linear model adjusting for the value at baseline as a covariate. PW, plain water; HW, H_2_-rich water.

At the baseline, there was no significant difference between two groups in the frequencies of apoptotic cells in the blood (*P* = 0.606) (Fig. 3). After the 4 week of trial, however, HW group showed a significantly lower percentage of PBMC apoptosis compared with PW group (*P* = 0.036) (Fig. 3).

Subsets of PBMCs were profiled with the antibodies specific for cell surface markers including CD4, CD8, CD20, CD14, and CD11b. PW and HW groups presented similar patterns of change in CD4^+^ (Δ = − 3.5 ± 4.8%, *P* < 0.01 in PW; Δ = − 2.4 ± 3.6%, *P* < 0.01 in HW), CD8^+^ (Δ = − 4.8 ± 2.1%, *P* < 0.001 in PW; Δ = − 4.5 ± 2.6%, *P* < 0.001 in HW) and CD11b^+^ cells (both *P* > 0.05) (Table 3). Although the frequency of CD20^+^ cell increased in the HW group compared to baseline values (Δ = 1.5 ± 2.5% and *P* < 0.05), there were no significant differences between the HW and PW groups (*P* = 0.900) (Table 3). It is notable that the change in the frequency of CD14^+^ cells in the HW group was significantly different from the change in the PW group (*P* = 0.039) (Table 3)**.**

**Table 3:** Percentages of peripheral blood immune cell subsets.

Cell type (%)	PW group (*n* = 18)			HW group (*n* = 19)			PW versus HW
	Baseline	Week 4	∆	Baseline	Week 4	∆	*P*
CD4	40.2 ± 9.0	36.7 ± 10.2	– 3.5 ± 4.8**	38.2 ± 7.3	35.8 ± 8.6	– 2.4 ± 3.6**	0.443
CD8	30.9 ± 6.6	26.1 ± 7.0	– 4.8 ± 2.1***	32.2 ± 7.4	27.8 ± 7.2	– 4.5 ± 2.6***	0.642
CD20	10.0 ± 3.2	11.0 ± 3.2	1.1 ± 3.0	9.0 ± 2.0	10.5 ± 2.9	1.5 ± 2.5*	0.900
CD14	7.2 ± 3.6	10.2 ± 10.7	2.9 ± 11.9	5.6 ± 2.3	5.0 ± 2.9	– 0.6 ± 3.5	0.039
CD11b	34.1 ± 6.5	36.8 ± 12.3	2.7 ± 13.6	31.6 ± 6.1	33.7 ± 10.4	2.1 ± 8.7	0.634

All values are means ± SDs. Percentages indicate the percent of live cells expressing the indicated cell surface markers. There were no significant differences between PW and HW groups for all immune cell frequencies at baseline on the basis of an unpaired *t* test or a Mann–Whitney *U* test. ∆ indicates the change from baseline to week 4. Significant differences between baseline and week 4 within each group were determined with a paired *t* test (*P < 0.05; **P < 0.01; ***P < 0.001).* P* values were obtained with the use of a general linear model adjusting for the value at baseline as a covariate. PW, plain water; HW, H_2_-rich water.

### Transcriptome profiles of PBMCs

**Figure 4:**
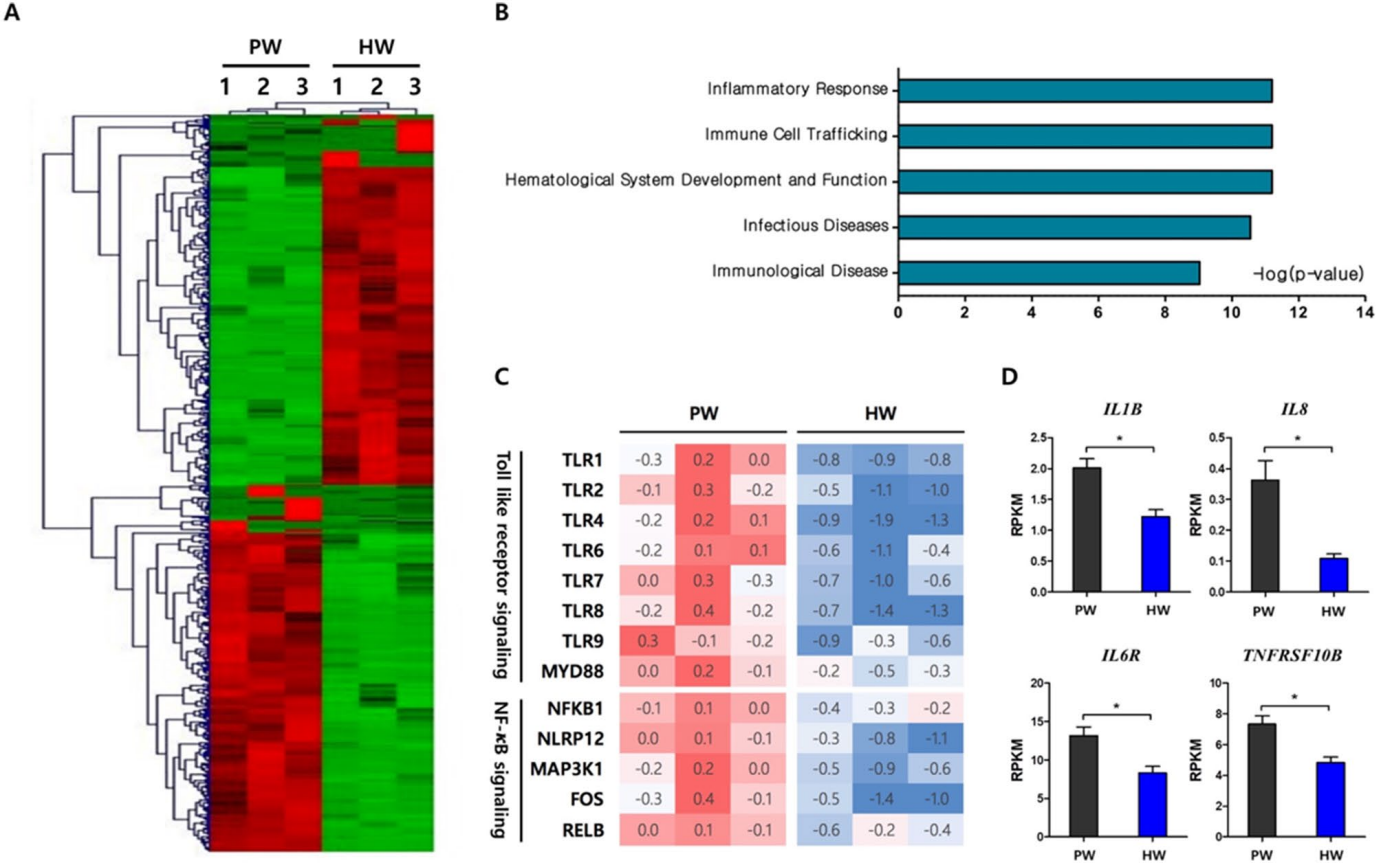
Transcriptome profiles of peripheral blood mononuclear cells at week 4. (**A**) Hierarchical clustering analysis of DEGs (**B**) Top 5 biological functional categories were discovered within DEGs by IPA. Statistical significance was calculated by the Fisher’s exact test and noted as a log (*P*-value). (**C**) Heat maps of expression levels of key genes related to toll like receptor and NF-κB signaling (**D**) HW group (*n* = 3) presented the lower expression levels in IL6R and NF-κB responsive genes including IL1B, IL8 and TNFRSF10B, compared with PW group (*n* = 3). Data are presented as means ± SEMs. Significant differences between PW and HW groups were determined with the use of an unpaired *t* test. PW, plain water; HW, H_2_-rich water; DEG, differentially expressed genes; IPA, Ingenuity Pathway Analysis; RPKM, reads per kilobase million.

In order to elucidate molecular mechanisms by which hydrogen-rich water consumption affects the apoptosis and immune cell profiles of PBMC, RNA-sequencing analysis in a genome-wide scale was carried out using total sets of RNAs from 6 individuals that included three randomly selected samples per group. A total of 605 differentially-expressed genes (DEGs) between the HW and PW groups were identified as described in “Methods”. Hierarchical clustering analysis showed transcriptomes of HW were readily distinguishable from those of PW (Fig. 4A). To gain insights into functional implications of the altered gene expression profiles caused by hydrogen water, the DEGs were categorized by physiological functions and a significance of the enrichment of each category was tested by Fisher’s exact test. Interestingly, the top 5 significant categories were Inflammatory response, Immune cell trafficking, Hematological system development and function and Infectious diseases and immunological disease (Fig. 4B). Within the top significant category, Inflammatory response, it was of interest that genes involved in TLR- NF-κB signaling were greatly reduced in expression. They included a series of toll-like receptors and key mediator molecules such as TLR1, TLR2, TLR4, TLR6, TLR7, TLR8, TLR9 and MYD88. In addition, transcription of intracellular proteins involved in NF-κB signaling including NFKB1, NLRP12 and MAP3K1 and, therefore, down-stream genes such as FOS and RELB were significantly reduced in the HW group (Fig. 4C). Also, we investigated the expression levels of genes responsive to NF-κB activation and those encoding pro-inflammatory cytokines and their receptors. Consequently, we observed that the HW group had the significantly lower expression levels in IL1B, IL8, IL6R, and TNFRSF10B than the PW group (Fig. 4D).

## Discussion

The effects of H_2_-rich water on antioxidant system have been tested largely within in vitro or animal models, with limited human data from few patient studies allowing substantiation of the beneficial roles of the water^19–21^. To the best of our knowledge, this is the first randomized clinical trial investigating the antioxidant activities of H_2_-water in heathy subjects, especially through a comprehensive analysis of oxidative stress markers, blood immune cell profiles, and the genome-scale gene expression. Four-week consumption of H_2_-water induced a substantial increase in the antioxidant capacity and a decrease in oxidative stress of DNAs, although there was no significance found in the comparison of an intervention (H_2_-water) and the placebo (plain water) group. These observations that H_2_-water showed some potential to have antioxidant activity, prompted us to further examine the effect on the apoptosis of peripheral blood cells in each subject, since even small changes in oxidative stress might be sufficient enough to initiate the apoptotic process. We found that the frequencies of apoptotic cells were significantly reduced by H_2_-water. In addition, flow cytometry analysis of peripheral blood showed that H_2_-water significantly reduced frequencies of circulating CD14^+^ cells. Interestingly, RNA-sequencing analyses identified a transcriptional network of inflammatory response as the most significant biological function modulated by H_2_-water. It greatly suppressed the expressions of genes involved in TLR-NF-κB signaling, as a result, the transcript levels of pro-inflammatory cytokines were significantly decreased.

There is extensive experimental evidence that oxidative stress can disrupt the cellular function by deforming the nucleic acids^22^; the oxidative DNA damage can be cytotoxic or mutagenic, and has been related to disease pathogenesis^23^. One of the most predominant forms of endogenous DNA lesion is 8-OHdG, which is formed by the addition of hydroxyl radical to the deoxyguanosin^24^. Thus, 8-OHdG has been widely used as a hallmark of oxidative stress and elevated levels of 8-OHdG might be a risk factor for cancer, atherosclerosis, and diabetics^25^. It is noteworthy that the concentration of 8-OHdG decreased to 35% of the baseline levels in the HW group, albeit no significance was found because of the 52% reduction in the PW group as well. Ishibashi et al*.* also observed that patients with rheumatoid arthritis showed a significant reduction in the levels of urinary 8-OHdG after intake of 530 mL/d of H_2_-water for 4 weeks^17^. The hydrogen-water has been shown to reduce DNA oxidation in animal model studies. H_2_-rich water treatment to rats inhibited an age-dependent increase in serum 8-OHdG levels^26^. The protective effect of H_2_ against DNA oxidative injury was also enhanced in a rabbit model of steroid-induced osteonecrosis, as revealed by quantifying 8-OHdG-positive haematopoietic cells^27^. Similarly, an intraperitoneal injection of H_2_-rich saline to rats was effective in decreasing the number of 8-OHdG-positive myocardial cells after inducing cardiac I/R injury^28^. One possible mechanistic explanation for the suppressive effect of HW on the production of 8-OHdG is that inert hydrogen reacts with hydroxyl radical^9^. However, further mechanistic studies are needed to identify whether a direct interaction between the hydroxyl radical and hydrogen exists when molecular hydrogen is administered via oral ingestion of H_2_-rich water. Unlike 8-OHdG, many varied molecules are involved in lipid peroxidation, including peroxy, alkoxy, alkyl radicals, ozone, and sulfur dioxide as well as the hydroxyl radical^23^. It has been known that H_2_ selectively removes the hydroxyl radicals without affecting other ROS^29^; thus, it is not surprising that no changes in d-ROMs were observed during the intervention.

Aging is generally characterized by a state in which systemic oxidative stress is elevated and/or the antioxidant defense system is altered, indicating dysregulation of redox balance and the accumulation of oxidative damages^30^. We therefore assumed that the effects of H_2_-water might vary with the age of participants. Although there was no difference in serum biological antioxidant potential between the intervention and placebo groups in the population as a whole, stratifying for age showed a significant increase in antioxidant capacity in the older group aged ≥ 30 y. The younger age group (< 30 y) showed no difference between H_2_-water and placebo groups. This finding implies that H_2_-water could exert antioxidant capacity-promoting benefits more in older adults than in the young.

Apoptosis is one of the consequences resulting from excessive ROS generation^31^. As the mitochondrial respiratory chain is the major source of endogenous ROS, mitochondrial DNA, proteins and lipids are susceptible to ROS attack, and these biomolecular damages beyond the capacity of repair can lead to programmed cell death^32^. Excessive destruction of normal cells constitutes a major cause of aging^33^, diabetes^34^ and neurodegenerative diseases^35^. Surprisingly, the HW group showed a lower percentage in apoptotic PBMC at week 4 compared with the PW group. This suggested that HW consumption was effective in preventing severe cellular damages. Because hydrogen molecules have small size and low molecular weight enough to diffuse across the cellular membrane and enter intracellular compartments, H_2_ may have directly suppressed these severe damages^36^. The anti-apoptotic function of H_2_ has been reported by others in animal model studies such as ischemia/reperfusion-induced rats^37^ and hypoxia–ischemia rats^38^. In addition, a human study of an uncontrolled clinical trial in patients with potential metabolic syndrome demonstrated an anti-apoptotic effect of HW consumption in endothelial cells^16^. The decrease in apoptosis in the present study may have been linked to the decrease in the frequency of CD14 positive PBMCs. CD14 is mainly expressed on the surface of human circulating monocytes^39^. Oxidatively-stressed cells induce CD14^+^ monocytes to migrate around them for apoptotic cell clearance^40^, and recruited monocytes successfully phagocytose the dying cells^41^. Thus, alleviation of oxidative stress resulted in a decrease in cell damage, which, in turn, decreased the frequency of circulating monocytes.

Oxidative stress and inflammation are tightly linked each other. Immune cells are stimulated by the ROS-damaged biomolecules to promote an inflammatory response^3,4^. Some ROS directly activate redox-sensitive proteins and transcription factors including mitogen-activated protein kinase (MAPK) and NF-κB. They also trigger the production of pro-inflammatory cytokines including IL-1 and IL-6^42,43^. Inflammatory cells generate ROS, thereby further enhancing these responses. Hydroxyl radicals act as a strong messenger for NF-κB activation which is pivotal in inflammation, consequently, radical-scavenging contributes to anti-inflammatory effects^44^. As shown in the present study, H_2_-water consumption remarkably down-regulated the NF-κB signaling pathway. H_2_ also suppressed NF-κB-regulated genes in the healthy mouse liver^45^. In animal studies with models of inflammation, H_2_-administration effectively decreased the levels of pro-inflammatory cytokines such as IL-1, IL-6, and TNF-α^46–49^. In addition, H_2_ has been reported to generate modified phospholipid, an antagonist of oxidized phospholipids, resulting in a decline in Ca^2+^ signaling and the Ca^2+^-dependent nuclear factor of activated T cells (NFAT) pathway which induces the production of pro-inflammatory cytokines^50^.

In the present study, 1.5 L of water was consumed daily by all participants whether they were in the intervention or placebo groups. Based upon an individual’s self-records on habitual water intake that was analyzed before participation, this intervention study prompted participants to drink 300 mL more water on average compared to their usual intake. Therefore, this increment in water intake might generate beneficial effects on the physiology of immune system, which might be attributable to the observation that biological antioxidant capacity was enhanced and oxidative DNA damage was reduced even by plain water. Some limitations of the study include a relatively short-term intervention, and thus the results cannot address the long-term effect of H_2_-water. In addition, study participants were mostly recruited from the Seoul National University and local residents, and therefore may not be representative of the general population of healthy adults. Finally, the study population number may have been not large enough to yield significant difference in blood oxidative stress markers.

In conclusion, this work presents, to our knowledge, the first double-blind placebo-controlled comprehensive study investigating the effects of H_2_-water in healthy adults. 1.5 L of H_2_-water intake for 4 weeks reduced cell death and inflammatory responses by modulating transcriptional networks of TLR-NFκB signaling. In addition, it may promote biological antioxidant capacity for adults > 30 yrs more than younger individuals.

## Methods

### Participants

158 individuals were recruited to the study which was advertised on the school portal website and bulletin boards. They were assessed for eligibility according to the following inclusion criteria: men and women aged 20–59 y; no medical history of acute or chronic diseases; and average daily consumption of water ranging from 500 to 2,500 mL. Exclusion criteria were as follows: consumption of beverages including coffee, tea, soft drinks and alcohol > 500 mL per day; consumption of alcohol containing beverages > 2 days per week; regular use of antioxidant supplements including vitamins and minerals within the last 3 months; and habits of smoking or strenuous exercise. A total of 117 volunteers were excluded based on the following reasons: 36 persons did not match our consumption standard of pure water (500–2,500 mL/day); 35 persons were consuming extra beverages (not pure water) over 500 mL/day; 29 persons had a history of regular use of antioxidant supplements within the last 3 months; 7 persons had a smoking habit; and 17 persons had a high level of physical activity according to International Physical Activity Questionnaire.

### Study design

This study was a 4-week, parallel-designed, randomized, double-blind, and placebo-controlled trial. Eligible participants were randomly assigned to either a plain water group (PW group) or a H_2_-rich water group (HW group), and the random assignment was stratified by sex and age (< 30 y and ≥ 30 y) with the use of an online randomization service (Sealed Envelope, London, UK). At baseline and after the trial, blood samples were collected when the participants were at rest. Participants in both the PW and HW arms were advised to maintain their usual diet and physical activities and to avoid taking any antioxidant supplements throughout the experimental period. All investigators and staffs involved in the random assignment, measurement and assessment of outcomes were blinded to the allocation. This study was conducted at the Department of Food and Nutrition in Seoul National University between Aug and Oct 2016, and was approved by the Institutional Review Board of Seoul National University (IRB No. 1606/001-012). All methods were performed in accordance with the relevant guidelines and regulations. This trial was registered at the Clinical Research Information Service (CRIS) on April 12th, 2019 (Registry No. KCT0003763). Written informed consent was provided by all participants prior to inclusion in the study.

### Water intervention

Commercially available H_2_-rich water (Koreahydrogenwater Corp., Seoul, Korea) and plain water (Coway Co., Ltd, Seoul, Korea) were used. The hydrogen concentration of the H_2_-rich water was 0.753 ± 0.012 mg/L when measured using the dissolved H_2_ analyzer (Orbisphere 3,654 portable analyzer; Hach, Switzerland, Geneva). A label was attached to each container and only information, such as the participant’s code and the date of manufacturing were provided on it. Each participant was provided daily with 3 bottles of 500 mL water, either PW or HW. All participants were instructed to finish the 500 mL of water bottle within an hour after opening the bottle to minimize a loss of dissolved H_2_. They were not allowed to drink any other additional water with an exception of coffee, tea, soft drinks and alcoholic beverages, but the total consumption of such extra drinks was controlled to ≤ 500 mL per day to minimize the variation in total beverage consumption. Participants were encouraged to record a daily history of water consumption, and any extra beverages if ever consumed. The records were reviewed 2 times a week to enhance their compliance to the study. The average compliances (%) for the HW group and the PW group were 99.2 ± 1.7 and 99.3 ± 1.1, respectively, with no statistical difference between the two groups (*P* = 0.762), as determined by Mann–Whitney *U* test. The analysis of extra beverage consumption showed no statistical difference between the two groups (HW group: 159.0 ± 82.0 mL/day; PW group: 143.0 ± 60.1 mL/day; *P* = 0.090, by an unpaired *t* test).

### Blood sampling

The first visit took place on the day before starting the intervention, and the second one was done on the day subsequent to the last day of the intervention. On each visit, participants filled in a questionnaire containing the questions about daily dietary intake and physical activities. Fasting venous blood samples from antecubital fossa were collected into 8-mL serum separator tubes (BD Biosciences, Franklin Lakes, NJ, USA), 8-mL EDTA-containing tubes (BD Biosciences), and BD vacutainer mononuclear cell preparation tubes with sodium citrate (BD Biosciences). Upon collection, plasma and serum samples were aliquoted in 1.5 mL ep-tubes (Eppendorf, Hamburg, Germany) and were frozen at − 80 °C for a later analysis.

### Measurements

- Antioxidant capacity and oxidative damages
  Antioxidant capacity was determined by measuring BAP in serum using a BAP test (BAP Kit; Diacron Srl., Grosseto, Italy). Oxidative stress in serum was assessed by the level of ROS-derived hydroperoxides measured using a diacron reactive oxygen metabolites kit (Diacron Srl.). 8-OHdG, an indicator of DNA damage by oxidative stress, was measured in serum with the use of an enzyme-linked immunosorbent assay (8-OHdG Check ELISA; Jaica, Fukuroi, Japan) in accordance to the manufacturer’s instructions.
- Apoptosis of PBMCs
  Annexin V staining was performed using PE-conjugated anti-annexin V antibody (eBioscience) in annexin V binding buffer (10 mM HEPES [pH7.4], 140 mM NaCl. 2.5 mM CaCl_2_) at RT for 15 min. DAPI (4′,6-diamidino-2-phenylindole; Sigma-Aldrich) staining was used for excluding dead cells and apoptotic analysis. Frequencies of apoptotic cells were analyzed using BD LSRFortessa (BD Biosciences, San Jose, CA, USA).
- Immune cell population profiles
  PBMCs were isolated from the whole blood by density-gradient centrifugation using Ficoll-Paque PLUS density gradient media (GE healthcare, Songdo, Korea). PBMCs were stained with Alexa Fluor 488-conjugated anti-human CD4 (OKT4; eBioscience, San Diego, CA, USA), PE-conjugated anti-human CD8 (3B5; eBioscience), APC-Cy7-conjugated anti-human CD20 (B-Ly-1; eBioscience), APC-Cy7-conjugated anti-human CD11b (ICRF44; BD Biosciences, San Jose, CA, USA), APC-conjugated anti-human CD14 (61D3; eBioscience) antibodies in FACS buffer (0.1% bovine calf serum and 0.05% sodium azide in 1 × PBS [phosphate buffered saline]) at 4 °C for 30 min. Profiles of each populations were analyzed by flow cytometry with FlowJo software (TreeStar, Ashland, OR, USA).
- Transcriptome profiles of PBMCs–RNA-next generation sequencing
  PBMCs were isolated immediately after the blood collection with the use of BD vacutainer mononuclear cell preparation tubes with sodium citrate (BD Biosciences) and then total RNA was extracted from PBMCs (RNAqueous-4PCR Kit; Ambion, TX, USA). Quality and concentration of extracted total RNA were assessed using Agilent 2,100 Bioanalyzer (Agilent Technologies, CA, USA). Out of the samples with RNA integrity number (RIN) greater than 8, a total of 6 samples (3 samples per a group) were randomly selected to be sequenced. Subsequently, intact mRNA was captured from the total RNA with the use of Dynabeads mRNA DIRECT Micro Kit (Ambion). Total mRNA samples were depleted of 5S, 5.8S, 18S, and 28S ribosomal subunits up to 99.9% using RiboMinus Eukaryote System v2 (Life Technologies, Carlsbad, CA, USA). Absence of ribosomal peaks was confirmed using Bioanalyzer and RNA 6,000 Pico Kit (Agilent Technologies). Barcoded cDNA libraries were prepared from the ribo-depleted mRNA samples and constructed with the use of reagents in Ion Total-RNA Seq Kit v2 (Life Technologies). First, the mRNA was fragmented with RNase III at 37 °C for 3 min. The fragmented RNA was purified on nucleic acid-binding beads and hybridized with Ion Adaptor Mix v2. Subsequently, ligation was performed at 30 °C for 1 h. The adaptor-ligated libraries were pre-incubated with a reverse transcription primer at 70 °C for 10 min and then converted to cDNA by reverse transcription at 42 °C for 30 min. The cDNA libraries were purified on nucleic acid-binding beads and then amplified by PCR using barcoded primers (Ion Xpress RNA-Seq Barcode 01–16 Kit; Life Technologies). After the bead-purification, molarity of the final library was determined using Bioanalyzer and High Sensitivity DNA Kit (Agilent Technologies). Whole transcriptome libraries were diluted to 100 pM using Bioanalyzer and amplified on Ion Sphere Particles (ISPs) by emulsion PCR with the use of Ion One Touch 2 system (Life Technologies) and Ion PI Hi-Q OT2 200 Kit (Life Technologies). Enrichment of template-positive ISPs were performed using Ion OneTouch Enrichment System (ES) (Life Technologies) where biotinylated adaptor sequences were selected by binding to streptavidin beads. Subsequently, the template-positive ISPs were sequenced with the use of Ion PI Hi-Q Sequencing 200 Kit (Life Technologies). Sequencing primers were annealed to the template fragments attached to ISPs, and the template positive ISPs samples were loaded on a chip of Ion PI Chip Kit v3 (Life Technologies) and incubated with polymerase. Finally, the chip was placed on Ion Proton System (Life Technologies) for sequencing working on the principal that hydrogen ion release was detected when new nucleotides were incorporated into the growing DNA template^51^. All procedures were performed according to the manufacturer's instructions.
- Bioinformatics analysis of RNA sequences
  Raw reads generated by the sequencer were trimmed and filtered. Trimming was performed to remove the adapter sequence and lower-quality 3′ ends with low quality scores. Read filtering was carried out to remove adapter dimers, reads lacking a sequencing key and polyclonal reads. High quality reads were mapped and aligned with the computational pipeline of Bowtie 2 and TopHat^52^. After mapping and aligning, the resulting BAM files were imported into Partek Genomics Suite v6.6 (Partek Inc., Saint Louis, MI, USA) and converted into gene transcript levels as reads per kilobase of exon per million mapped reads (RPKM) with the use of a mixed-model approach. DEGs were identified with a fold-change threshold (greater than 2 or less than − 2) and *P* value (*P* < 0.01). Genes that passed our statistical criteria were analyzed with the bioinformatics software Ingenuity Pathway Analysis (IPA; www.ingenuity.com). Hierarchical clustering analysis and biological classification analysis was performed. Fisher’s exact test was used to test a significance for the enrichment of specific biological processes in the set of DEGs.

### Statistical analysis

Statistical analysis was performed with the use of SPSS version 23 for Macintosh (IBM Corp., Chicago, IL, USA). A sample size was calculated based on a previous study^17^ with an α = 0.05 and a power of 80%. All data were tested for normality before selecting the appropriate statistical method. General characteristics at baseline were analyzed on the basis of an unpaired *t* test or Mann–Whitney *U* test to identify whether there were statistical differences between groups. A paired *t* test or a Wilcoxon signed-rank test was used for within-group comparisons between baseline and week 4. The changes from baseline to week 4 were compared between PW and HW groups on the basis of a general linear model with an adjustment for the value at baseline as a covariate. We conducted a two-way ANOVA with an adjustment for the value at baseline as a covariate to determine the interaction between the effects of treatment (PW or HW) and age (< 30 y or ≥ 30 y) regarding the changes from baseline to week 4 in BAP, d-ROMs, 8-OHdG, PBMC apoptosis and subsets. When a significant interaction was discovered, simple main effects analysis was performed. *P* < 0.05 was considered statistically significant.

## Data Availability

The data that support the findings of this study are available from the corresponding author upon reasonable request.
